# Genomic Classification and Clinical Outcome in Rhabdomyosarcoma: A Report From an International Consortium

**DOI:** 10.1200/JCO.20.03060

**Published:** 2021-06-24

**Authors:** Jack F. Shern, Joanna Selfe, Elisa Izquierdo, Rajesh Patidar, Hsien-Chao Chou, Young K. Song, Marielle E. Yohe, Sivasish Sindiri, Jun Wei, Xinyu Wen, Erin R. Rudzinski, Donald A. Barkauskas, Tammy Lo, David Hall, Corinne M. Linardic, Debbie Hughes, Sabri Jamal, Meriel Jenney, Julia Chisholm, Rebecca Brown, Kristine Jones, Belynda Hicks, Paola Angelini, Sally George, Louis Chesler, Michael Hubank, Anna Kelsey, Susanne A. Gatz, Stephen X. Skapek, Douglas S. Hawkins, Janet M. Shipley, Javed Khan

**Affiliations:** ^1^ Genetics Branch, Oncogenomics Section, Center for Cancer Research, National Institutes of Health, Bethesda, MD; ^2^ Pediatric Oncology Branch, Center for Cancer Research, National Institutes of Health, Bethesda, MD; ^3^ Sarcoma Molecular Pathology Team, Divisions of Molecular Pathology and Cancer Therapeutics, The Institute of Cancer Research, London, United Kingdom; ^4^ Molecular Diagnostics Department, The Institute of Cancer Research and Clinical Genomics, The Royal Marsden NHS Foundation, London, United Kingdom; ^5^ Department of Laboratories, Seattle Children's Hospital, University of Washington, Seattle, WA; ^6^ Department of Preventive Medicine, Keck School of Medicine of the University of Southern California, Los Angeles, CA; ^7^ Children's Oncology Group, Monrovia, CA; ^8^ Duke University School of Medicine, Durham, NC; ^9^ Paediatric Tumour Biology, Division of Clinical Studies, The Institute of Cancer Research, London, United Kingdom; ^10^ Cardiff and Vale UHB, Paeds Oncology, Cardiff, United Kingdom; ^11^ Children and Young People's Unit, Royal Marsden NHS Foundation Trust, London, United Kingdom; ^12^ Department of Pathology, Aberdeen Royal Infirmary, Aberdeen, United Kingdom; ^13^ Cancer Genomics Research Laboratory, Leidos Biomedical Research, Frederick National Laboratory for Cancer Research, Frederick, MD; ^14^ Department of Paediatric Histopathology, Manchester University NHS Foundation Trust Royal Manchester Childrens Hospital, Manchester, United Kingdom; ^15^ Cancer Research UK Clinical Trials Unit, Institute of Cancer and Genomic Sciences, University of Birmingham, Birmingham, United Kingdom; ^16^ Division of Hematology/Oncology, Department of Pediatrics, University of Texas Southwestern Medical Center, Dallas, TX; ^17^ Department of Pediatrics, Seattle Children's Hospital, Fred Hutchinson Cancer Research Center, University of Washington, Seattle, WA

## Abstract

Rhabdomyosarcoma is the most common soft tissue sarcoma of childhood. Despite aggressive therapy, the 5-year survival rate for patients with metastatic or recurrent disease remains poor, and beyond *PAX-FOXO1* fusion status, no genomic markers are available for risk stratification. We present an international consortium study designed to determine the incidence of driver mutations and their association with clinical outcome.

**PATIENTS AND METHODS:**

Tumor samples collected from patients enrolled on Children's Oncology Group trials (1998-2017) and UK patients enrolled on malignant mesenchymal tumor and RMS2005 (1995-2016) trials were subjected to custom-capture sequencing. Mutations, indels, gene deletions, and amplifications were identified, and survival analysis was performed.

**RESULTS:**

DNA from 641 patients was suitable for analyses. A median of one mutation was found per tumor. In *FOXO1* fusion-negative cases, mutation of any RAS pathway member was found in > 50% of cases, and 21% had no putative driver mutation identified. *BCOR* (15%), *NF1* (15%), and *TP53* (13%) mutations were found at a higher incidence than previously reported and *TP53* mutations were associated with worse outcomes in both fusion-negative and *FOXO1* fusion-positive cases. Interestingly, mutations in *RAS* isoforms predominated in infants < 1 year (64% of cases). Mutation of *MYOD1* was associated with histologic patterns beyond those previously described, older age, head and neck primary site, and a dismal survival. Finally, we provide a searchable companion database (ClinOmics), containing all genomic variants, and clinical annotation including survival data.

**CONCLUSION:**

This is the largest genomic characterization of clinically annotated rhabdomyosarcoma tumors to date and provides prognostic genetic features that refine risk stratification and will be incorporated into prospective trials.

## INTRODUCTION

Rhabdomyosarcoma (RMS) is the most common soft tissue sarcoma of childhood.^1^ With the development of multimodal chemotherapy regimens, relapse-free survival rates have improved to 70%-80% in patients with localized disease, albeit with significant toxicity.^2^ Unfortunately, despite aggressive therapy, the 5-year survival rate for patients with metastatic disease remains poor, but variable.^3^ Therapy assignment in North American and European trials is currently based on clinicopathologic features and not molecular or genetic markers, with the exception of the recent incorporation of *FOXO1* fusion status by the Children's Oncology Group (COG) and European paediatric Soft tissue sarcoma Study Group (EpSSG).^4–6^ Although clinical features reasonably stratify patients into broad treatment cohorts, prognostic imprecision hampers efforts to successfully escalate or de-escalate therapy. Particularly problematic is the COG intermediate risk category, defined as localized *FOXO1* fusion-positive (FP) RMS and localized, incompletely resected (clinical group III) *FOXO1* fusion-negative (FN) RMS arising from an unfavorable anatomic site; this category comprises approximately 50% of cases and has a heterogeneous clinical outcome.^4,7,8^ This suggests that some of these children could be treated with less aggressive therapy or alternatively should be considered to have more aggressive disease.

**CONTEXT**

**Key Objective**
Rhabdomyosarcoma (RMS) is a sarcoma of childhood with a poor 5-year survival rate for patients with metastatic or recurrent disease. No genomic markers are currently available for risk stratification except for *PAX-FOXO1* fusion gene status. This study performed sequencing to determine the mutational status of genes implicated in RMS oncogenesis and correlated these results with clinical outcomes.
**Knowledge Generated**
The genetic and clinical characteristics associated with primary tumors from two international cohorts are presented. Survival analysis demonstrated that *TP53* and *MYOD1* mutations were associated with worse event-free survival.
**Relevance**
This study nominates mutant genes *MYOD1* and *TP53* as indicators of poor prognosis in fusion-negative RMS, and *TP53* alterations as a biomarker of more aggressive disease in fusion-positive RMS. Mutation of *MYOD1* was not restricted to spindle histology, and the association with adverse outcome highlights the need to accurately diagnose *MYOD1* mutations and develop novel treatment strategies for these patients.

Previous comprehensive genomic sequencing studies of RMS have been completed, but outcome analysis was limited by sample size or incomplete clinical annotation of the included samples.^9,10^ To genetically classify RMS and refine risk stratification, we formed an international collaborative group and performed standardized sequencing of a large cohort of clinically annotated cases. Herein, we report the summary findings and detail the importance of incorporation of genomic data into prospectively enrolling RMS clinical trials. Adopting molecular features to RMS risk stratification should improve clinical outcomes for patients with RMS by allowing further tailoring of therapies to match an individual patient's risk and mutational profile. To ensure that this critical data set is available to the broader research community, the generated sequencing data are available within dbGAP (accession phs000720.v4.p1) and the clinical and mutational data are publicly accessible.^11^

## PATIENTS AND METHODS

### Study Population and Clinical Annotation

Samples from the two cohorts included in this work were collected on institutional review board–approved clinical trials or tissue banking studies. COG samples included samples collected on ARST0331, ARST0431, D9602, D9803, and D9902. UK samples from patients treated on protocols through the malignant mesenchymal tumor and RMS2005 protocols^12-14^ were collected and approved for study through local and national ethical approvals (CCR2015 and 06/MRE04/71, respectively) (Appendix Table A1, online only). Because there are subtle differences in risk stratification between the COG and EpSSG (reviewed in Ref. 15), an overarching simplified risk stratification definition was used to enable merging data. Additional population and annotation details are provided in the Data Supplement (online only).

### Gene Panel Sequencing

A custom-capture sequencing assay targeting 39 genes previously implicated in RMS (Appendix Table A2, online only) was performed, and variants were called using previously published sequencing algorithms.^9,16-19^ Detailed sequencing and variant calling methods are provided in the Data Supplement.

### Statistical Methods

Patient characteristics were summarized using medians and ranges or frequencies and percentages. Associations between pairs of gene markers were tested using the exact conditional test of proportions (Fisher's exact test). Event-free survival (EFS) was defined as time from diagnosis (United Kingdom) or enrollment on a study (COG) until event (relapse, second malignant neoplasm, or death) or last contact. Each gene predictor variable was tested for univariate association with EFS and overall survival using the log-rank test and for association within a multivariate Cox proportional hazard regression. The survival analysis was done separately for each cohort of patients: COG and United Kingdom and within each cohort, separately for FP and FN patients. Detailed statistical methods are provided in the Data Supplement.

## RESULTS

### Patient Population

**FIG A1.**
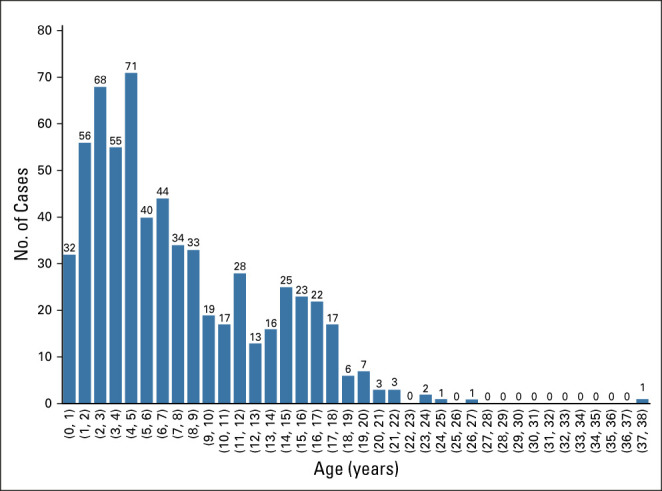
Histogram presentation of the age distribution of cases within the cohort (N = 641).

Clinically annotated cases from patient samples were assembled on COG biology study ARST14B1Q and a parallel cohort assembled from UK malignant mesenchymal tumor and RMS2005 studies, to generate two large cohorts of samples. The clinical details are summarized in Table 1. In total, 641 cases had adequate DNA to generate sequencing libraries of minimum quality to be included in the study. The median age of the combined cohort was 5.9 years (range 0.02-37.8) (Appendix Fig A1, online only). The male:female ratio was 1.9:1, slightly higher than the generally accepted ratio of 1.5:1,^20^ reflecting an enrichment of paratesticular tumors within this cohort. The most common anatomic locations were the parameningeal (20%) and paratesticular tumors (20%) followed by tumors of the retroperitoneum, peritoneum, or trunk (16%) and the extremity (14%). A simplified risk stratification algorithm was used to harmonize COG and UK cohorts (Data Supplement) and using this method, the population had representation of cases of the low-risk (34%), intermediate-risk (47%), and high-risk populations (18%).

**TABLE 1.**
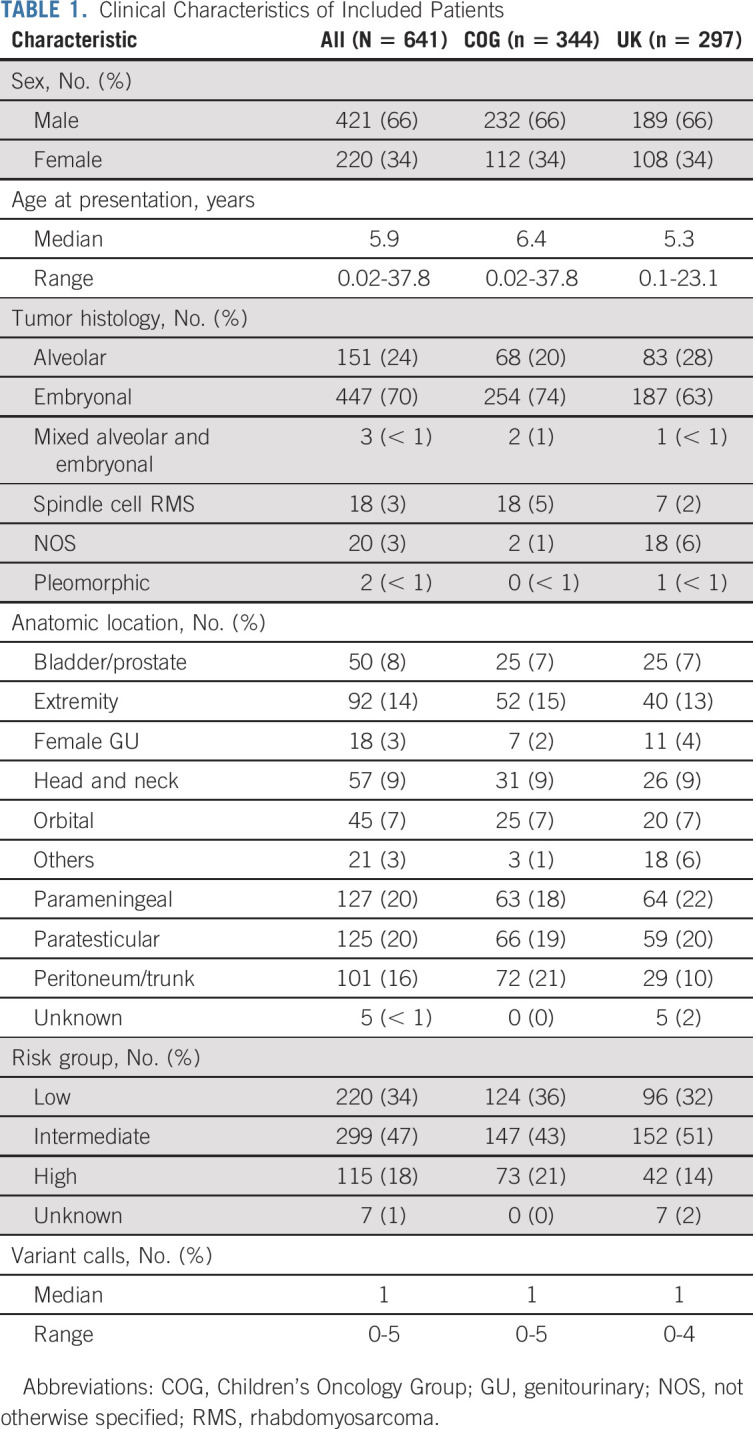
Clinical Characteristics of Included Patients

### Mutation Frequency Observations

**FIG 1.**
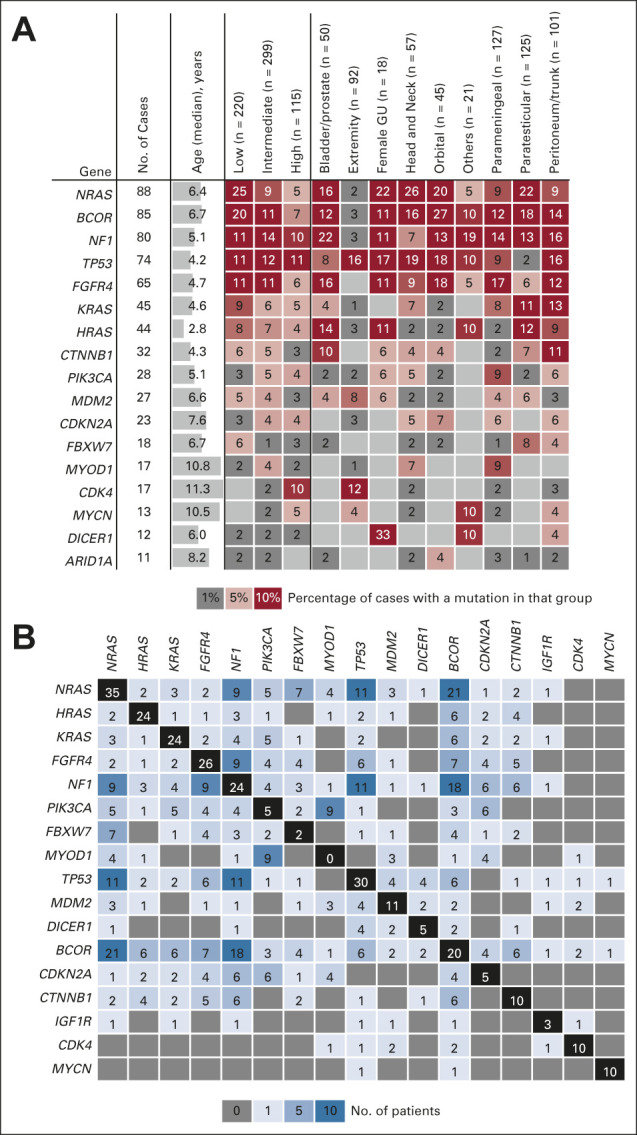
Mutations summarized by anatomic location and co-occurrence. (A) Summary of mutations by occurrence within defined risk groups (low, intermediate, or high) or by anatomic location. The reported value is a percentage of the number of cases with a mutation in that gene within each group. (B) Co-occurrence of mutated genes reported as absolute number of cases. GU, genitourinary.

Overall, the sequenced tumors had a median of one mutation call per tumor (range 0-5). The most frequently observed gene mutations are presented in Table 2. Consistent with prior reports, the genomic profiles of the FP and FN populations were distinct. The most frequently observed lesions in FP tumors were the focal amplification of *CDK4* (13%) or *MYCN* (10%). The genes *BCOR* (6%), *NF1* (4%), *TP53* (4%), and *PIK3CA* (2%) were found in a small number of FP RMS cases, verifying previous observations.^9^ In contrast, the most frequently observed genetic alteration in FN tumors were RAS isoform mutations *NRAS* (17%), *KRAS* (9%), and *HRAS* (8%), with any *RAS* isoform mutation noted in 32% (n = 167 of 515) of FN tumors. Mutation of an RAS pathway gene (defined as *NRAS*, *KRAS*, *HRAS*, *FGFR4*, *NF1*, and *PIK3CA*) could be found in 56% (n = 288 of 515) of all FN samples. Recurrence of mutations in tumor suppressor genes in FN RMS, *TP53* (13%), *NF1* (15%), and *BCOR* (15%), was higher than reports in previous studies.^9^ Hotspot mutations in *FGFR4* (13%), *CTNNB1* (6%), *PIK3CA* (5%), and *MYOD1* (3%) were observed at similar frequencies as previously reported and seen at similar percentages within the two independent international patient cohorts.^9,10^ No mutations were found in 14 genes previously associated with RMS (*MTOR*, *PKN1*, *ALK*, *SOS1*, *SOS2*, *ROBO1*, *PDGFRA*, *GAB1*, *BRAF*, *CCND1*, *CCND2*, *ATM*, *AKT*, and *SMARCA4*), although variants of unknown significance were observed in each of these genes. The median age of presentation of the patients correlated with alteration of individual genes, with a notable increase in *MYOD1* mutations, *CDK4* amplification, and *MYCN* amplification in patients older than 10 years and *HRAS* mutations in infants < 1 year (Fig 1A).

**TABLE 2.**
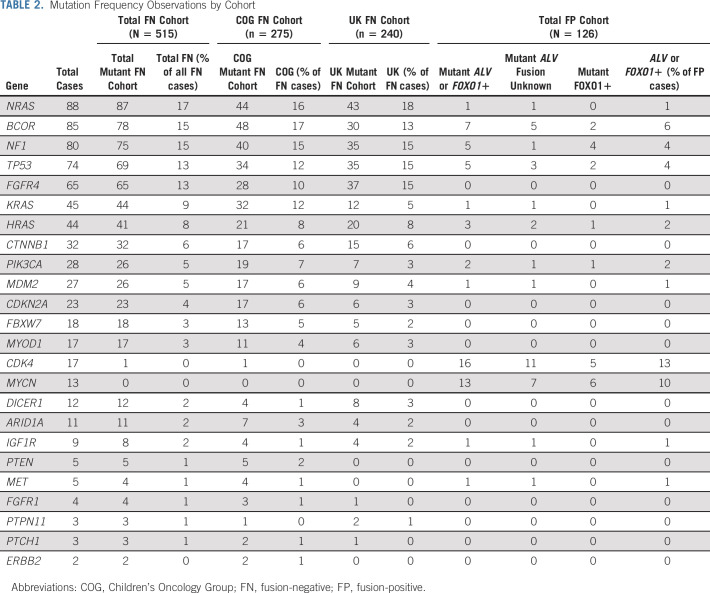
Mutation Frequency Observations by Cohort

### Mutations Summarized by Anatomic Distribution

RMS tumors arise in diverse anatomic locations throughout the body, and the site of disease is known to correlate with clinical outcome.^21^ Current clinical risk stratification assigns tumors arising in the orbit, nonparameningeal head and neck, and the male or female genital tracts as favorable. The distribution of mutations by anatomic location is presented in Figure 1A. As previously described,^22^ *DICER1* mutations had a predilection for tumors arising within the female genitourinary tract. Within the sequenced tumors, 33% (6 of 18) of all female genitourinary cases harbored a mutation in *DICER1*. Interestingly, FN cases of the extremity had an enrichment for *TP53* mutations or *MDM2* amplifications, whereby 42% (15 of 35) of FN cases with a primary tumor of the extremity had an alteration of one of these two genes (Appendix Table A3, online only). *MYOD1*-mutant tumors also showed a distinct anatomic enrichment with 88% (15 of 17) of *MYOD1*-mutant tumors observed in either the head and neck or parameningeal region.

### FN Tumors Frequently Harbor More Than One Genetic Driver Alteration

**FIG 2.**
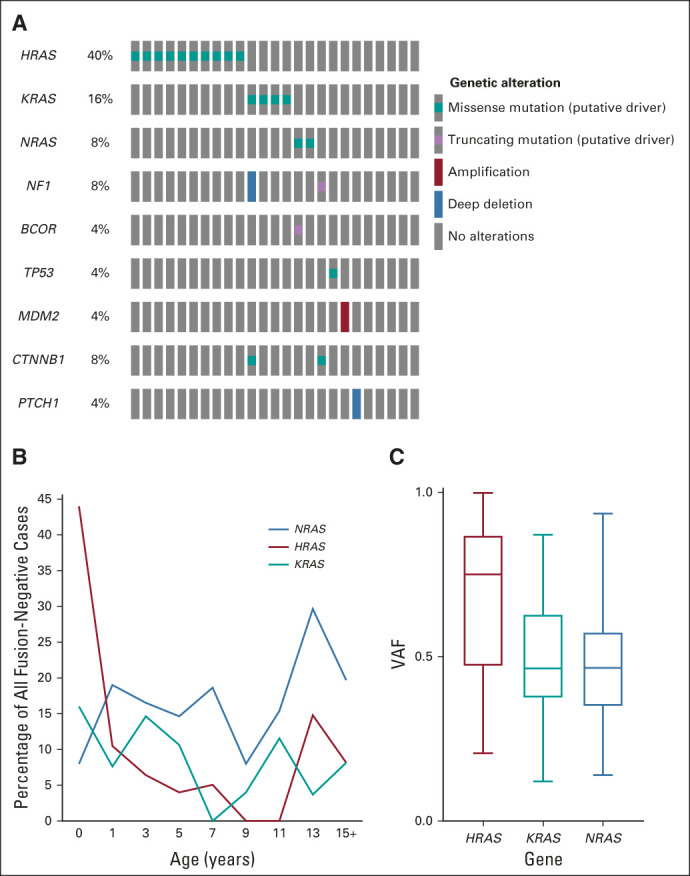
*RAS* isoform mutations. (A) Oncoprint of mutations observed in infants < 1 year old (n = 25) showed an enrichment for RAS mutations. (B) Distribution of RAS isoform mutations by age with a distinct peak of *HRAS* mutant cases discovered in the infant population. (C) *HRAS* mutations were frequently found to have a higher VAF indicating that the mutation occurred before a loss-of-heterozygosity event on chromosome 11p. VAF, variant allele frequency.

**FIG A2.**
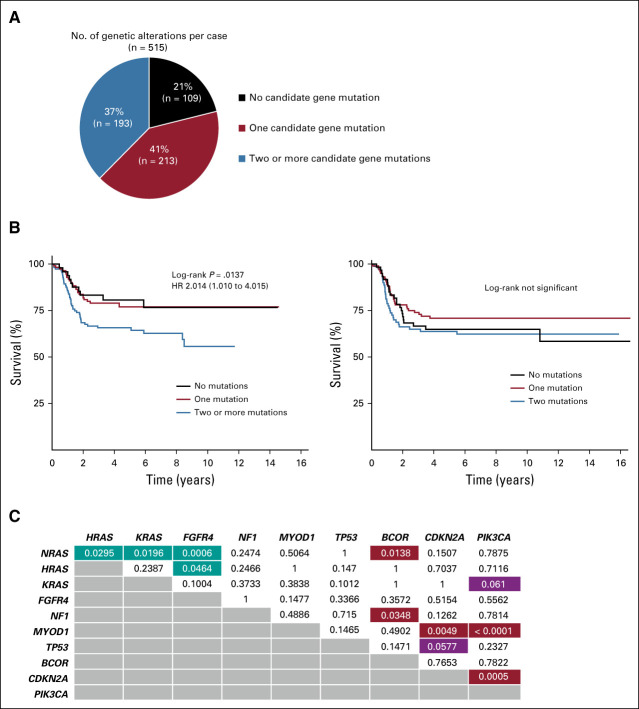
Survival analysis on the basis of number of called mutations. (A) All FN samples (n = 515) were distributed into groups having no observed mutations (black), one mutation (red), or two or more genes mutated (blue). (B) Kaplan-Meier analysis of EFS from the COG and UK FN cohorts on the basis of the number of observed mutations within a tumor. The COG cohort (n = 275) had 49 patients with no observed mutation, 113 patients with one observed mutation, and 113 patients with two or more observed mutations. The UK cohort (n = 240) had 60 patients with no observed mutation, 100 patients with one observed mutation, and 80 patients with two or more observed mutations. (C) Pairwise testing of the significance of gene-gene interactions was performed to identify interactions that occurred at higher (red and purple) or lower (green) frequencies than expected. COG, Children's Oncology Group; EFS, event-free survival; FN, fusion-negative; HR, hazard ratio.

Mutational heterogeneity has previously been described in FN RMS.^23^ In this study, the presence of multiple driver mutations within individual tumors was evident within FN samples with 41% (213 of 515) of tumors having one mutation, 37% (193 of 515) of tumors having two or more mutations, and 21% (109 of 515) containing no alteration of a candidate gene (Appendix Fig A2A, online only). Greater than two mutations within a tumor was a significant marker in terms of worse EFS (*P* = .01, hazard ratio [HR] 2.014 [1.010-4.015]) within the COG cohort; however, this observation was not replicated in the UK cohort for EFS (*P* = .39, HR 1.098 [0.633-1.904]) (Appendix Fig A2B). To establish the most common pairings of gene interactions that drive RMS, we analyzed mutational data across the cohort and observed that mutations of tumor suppressor genes such as *NF1*, *TP53*, and *BCOR* frequently co-occurred with other mutations. Significant interactions included *BCOR* with *NRAS* (*P* = .01) or *NF1* (*P* = .03), and *MYOD1* with *PIK3CA* (*P* < .0001) or *CDKN2A* (*P* = .0049) (Fig 2B, Appendix Fig A2C). *MYOD1* mutations were exceptional in that they always occurred with an additional gene being mutated. Although *NRAS*, *KRAS*, *HRAS*, and *FGFR4* were mutually exclusive in most cases, surprisingly, there were individual tumors with co-occurrence of multiple hotspot mutations in these genes.

### *CDK4*, *MYCN*, and *TP53* in FP Tumors

**FIG A3.**
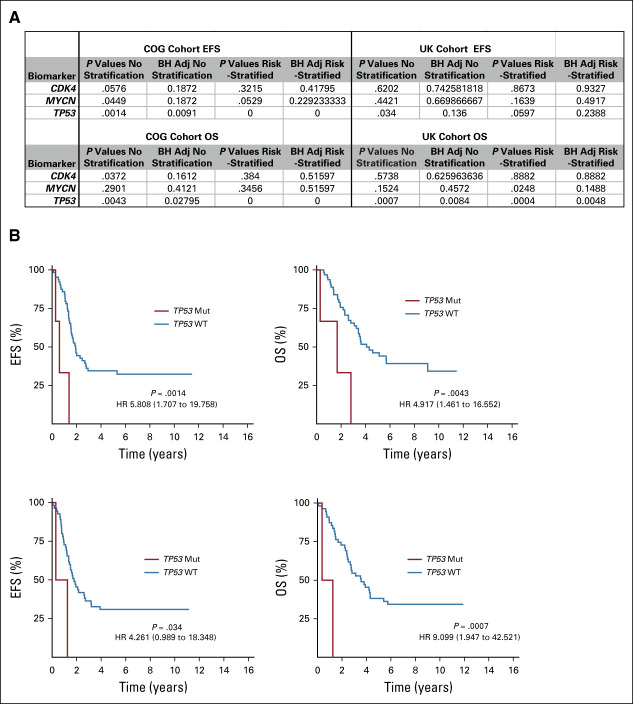
*CDK4*, *MYCN*, and *TP53* mutations in FP RMS. (A) Survival analysis for all three genes within the COG and UK cohorts with and without risk stratification. The log-rank and BH adj *P* value are presented. (B) Kaplan-Meier analysis of EFS and OS in FP cases within the COG cohort (n = 69) and UK cohort (n = 57) for *TP53* Mut or WT. COG cohort has three *TP53* mutant cases and UK cohort has two *TP53* mutant cases. BH adj, Benjamini-Hochberg–adjusted; COG, Children's Oncology Group; EFS, event-free survival; FP, fusion-positive; HR, hazard ratio; Mut, mutated; OS, overall survival; RMS, rhabdomyosarcoma; WT, wild type.

Although *PAX3-FOXO1* fusion is itself of prognostic value, no molecular markers are currently available for risk substratification of FP tumors. In total, 126 FP tumors were evaluated (69 COG and 57 UK) with a dedicated fusion assay performed on 80 of 126 (64%) and centrally reviewed alveolar histology used as a proxy in the remaining cases. Small numbers of *CDK4-* and *MYCN-*mutant cases (COG: n = 14 and n = 10; UK: n = 2 and n = 3, respectively) were observed between the two cohorts, limiting conclusions about the prognostic significance of these genes (Appendix Fig A3A, online only). Interestingly, a small number of FP cases (COG: n = 3; UK: n = 2) had mutations in *TP53* and were universally fatal (Appendix Fig A3B).

### Survival Analysis of RAS Isoforms and Enrichment of RAS Mutations in Infants

**FIG A4.**
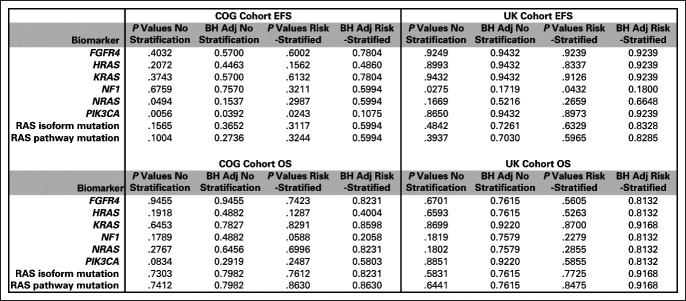
RAS isoform and RAS pathway analysis. (A) RAS isoforms and pathway member *P* values for both EFS and OS. The log-rank *P* value and the BH adj value are presented. BH adj, Benjamini-Hochberg–adjusted; COG, Children's Oncology Group; EFS, event-free survival; OS, overall survival.

**FIG A5.**
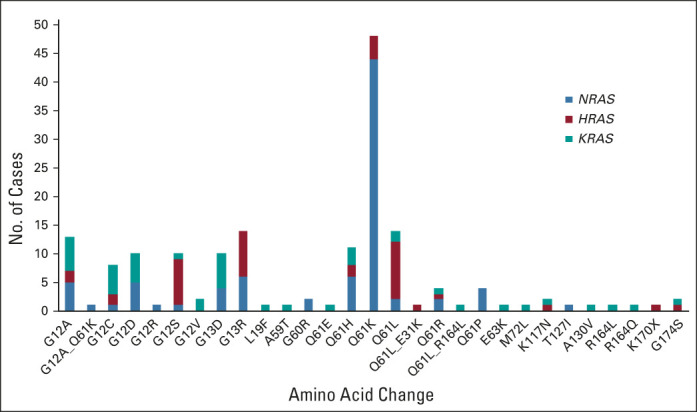
*RAS* codon and amino acid usage summary. Absolute number of cases with a mutation in the specified codon by amino acid change and isoform (*NRAS*, blue; *HRAS*, red; *KRAS*, green).

A driving hypothesis of this study was that the presence of a mutation in a RAS isoform or RAS pathway gene would correlate with poor outcomes in FN RMS, because of the observation in previous smaller cohorts of enrichment of RAS isoform mutations in high-risk cases.^10^ Therefore, we examined each RAS isoform and individual RAS pathway members for correlation with survival. Neither mutation of any RAS isoform nor a RAS pathway gene was associated with a worse EFS or overall survival across the two cohorts (Appendix Fig A4, online only). One striking observation was the correlation of RAS isoform mutations longitudinally with age. Most notable was the enrichment of RAS isoform mutations in FN cases that occurred under the age of 1 year (64% of cases, *P* = .0068) with a clear peak incidence of *HRAS* mutations (40% of all FN infants, *P* < .0001) within this age group and no enrichment of secondary mutations (Fig 2A). Although not significant, *KRAS*-mutant tumors were frequent within the toddler period (15% of cases at 3 years, *P* = .2368) and a peak of *NRAS*-mutant tumors was observed in adolescence (30% of all cases at 13 years, *P* = .4407) (Fig 2B). *NRAS*, *HRAS*, and *KRAS* isoforms had distinct codon and amino acid profiles, consistent with previous studies (Appendix Fig A5, online only). Interrogation of the observed allele frequency of RAS isoform mutations showed that the majority of *HRAS* mutations occur at variant allele frequency > 0.5, likely reflecting the occurrence of this mutation within the frequently observed uniparental disomy event that occurs on chromosome 11p (Fig 2C).^24^

### Association of *TP53* Alterations With Survival in FN Tumors

**FIG 3.**
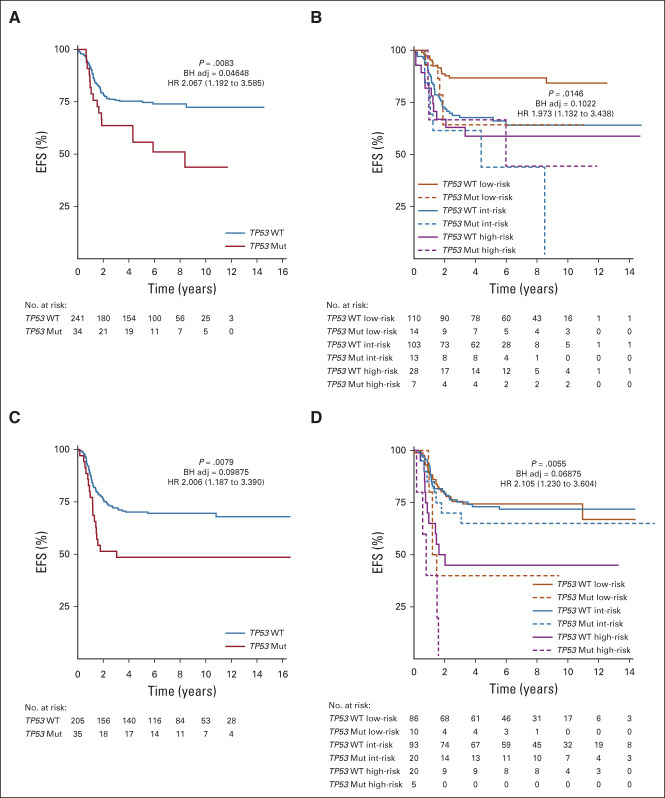
*TP53* mutations and association with survival. (A) KM analysis of EFS within the COG FN cohort (n = 275) by the presence of a *TP53* Mut or absence of a *TP53* lesion (*TP53* WT). (B) KM analysis of EFS within the COG FN cohort by *TP53* status and RMS risk category. Total case numbers: low, n = 124; intermediate, n = 126; high, n = 35. (C) KM analysis of EFS within the UK FN cohort (n = 240) by the presence of a *TP53* Mut or absence of a *TP53* lesion (*TP53* WT). (D) KM analysis of EFS within the UK FN cohort by *TP53* status and RMS risk category. Total case numbers: low, n = 96; intermediate, n = 113; high, n = 25. Presented *P* values are log-rank and BH adj. HR with 95% CI. BH adj, Benjamini-Hochberg–adjusted; COG, Children's Oncology Group; EFS, event-free survival; FN, fusion-negative; HR, hazard ratio; KM, Kaplan-Meier; RMS, rhabdomyosarcoma; *TP53* Mut, *TP53* mutation; *TP53* WT, *TP53* wild type.

**FIG A6.**
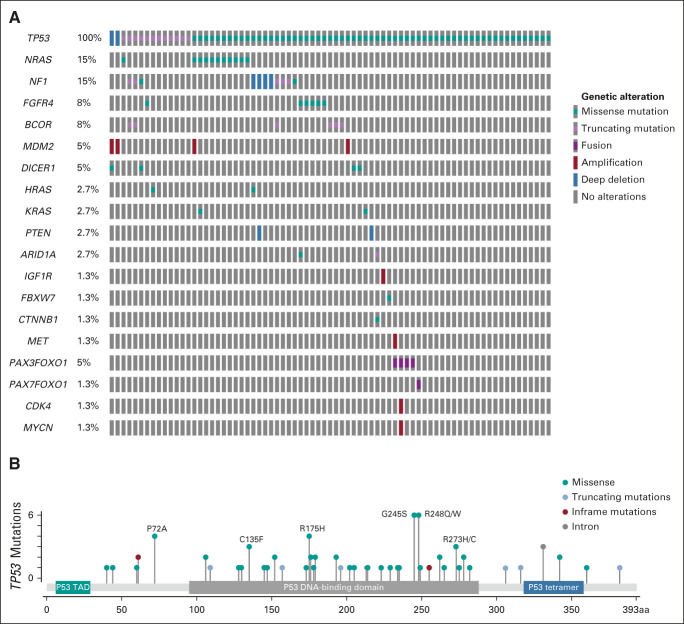
*TP53* mutations. (A) Association of *TP53* mutations with other gene mutations across the entire rhabdomyosarcoms cohort. (B) Location and number of mutations across TP53 protein.

*TP53* was found to be altered in 13% (n = 69 of 515) of the FN cohort, and the observed lesions included deep deletions, truncating mutations, and point mutations (Appendix Fig A6A, online only). The mutations occurred throughout the gene body with some enrichment seen within the DNA-binding domain of the protein (Appendix Fig A6B). The most recurrent mutations were found at the codons G245S (six cases), R248Q or W (six cases), R175H (four cases), and P72A (four cases). Given the lack of a matched normal sample, no determination was made if these lesions represent somatic or germline events. Univariate (EFS *P* = .0083; HR 2.067 [1.192-3.585]) and risk-stratified analysis (EFS *P* = .0146; HR 1.973 [1.132-3.438]) of survival data within the COG cohort demonstrated that the presence of a *TP53* mutation imparted a worse EFS (Figs 3A and 3B). Evaluation of the UK cohort verified the significance of this observation in both non–risk-stratified (EFS *P* = .0079; HR 2.006 [1.187-3.390]) and risk-stratified (EFS *P* = .0055; HR 2.105 [1.230-3.604]) analysis (Figs 3C and 3D).

### Association of *MYOD1* Mutations With Survival in FN Tumors

**FIG 4.**
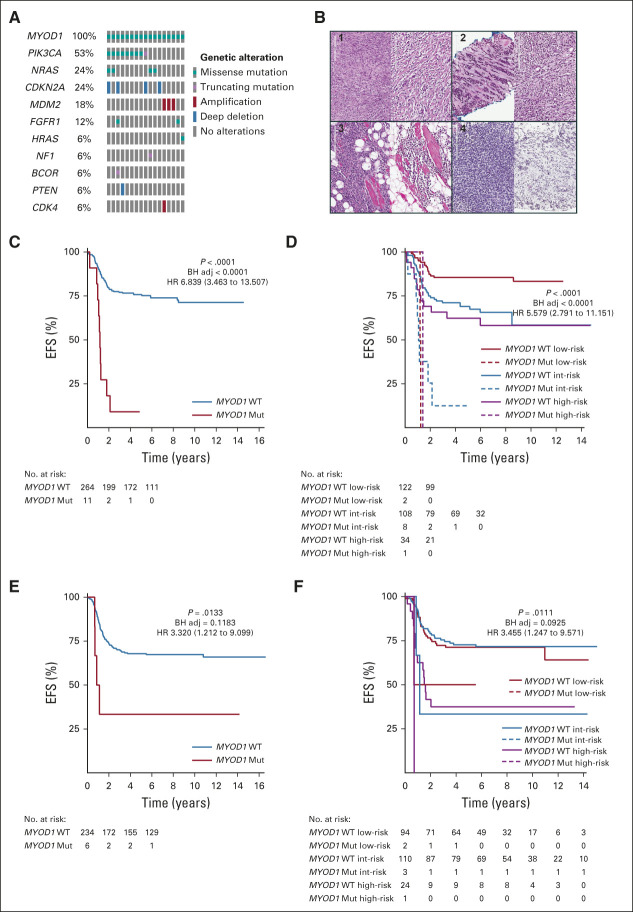
*MYOD1* mutations and survival. (A) All tumors with a mutation in *MYOD1* within the entire sequenced cohort (N = 641) are shown to visualize co-occurrence of *MYDO1* mutations with other genes. All *MYOD1* cases were observed within FN cases. (B) Histology of *MYOD1* mutant cases demonstrated the frequent presence of sclerosing or spindle morphology (case 1 and case 2). Cases 3 and 4 show that some cases demonstrated areas more typical of dense embryonal histology. (C) KM analysis of EFS within the COG FN cohort (n = 275) by the presence of a *MYOD1* Mut or absence of a *MYDO1* lesion (*MYOD1* WT). (D) KM analysis of EFS within the COG FN cohort by *MYOD1* status and RMS risk category. Total case numbers: low, n = 124; intermediate, n = 126; high, n = 35. (E) KM analysis of EFS within the UK FN cohort (n = 240) by the presence of a *MYOD1* Mut or absence of a *MYDO1* lesion (*MYOD1* WT). (F) KM analysis of EFS within the UK FN cohort by *MYOD1* status and RMS risk category. Total case numbers: low, n = 96; intermediate, n = 113; high, n = 25. Presented *P* values are log-rank and BH adj. HR with 95% CI. BH adj, Benjamini-Hochberg–adjusted; COG, Children's Oncology Group; EFS, event-free survival; FN, fusion-negative; HR, hazard ratio; KM, Kaplan-Meier; *MYOD1* Mut, *MYOD1* mutation; *MYOD1* WT, *MYOD1* wild type; RMS, rhabdomyosarcoma.

**FIG A7.**
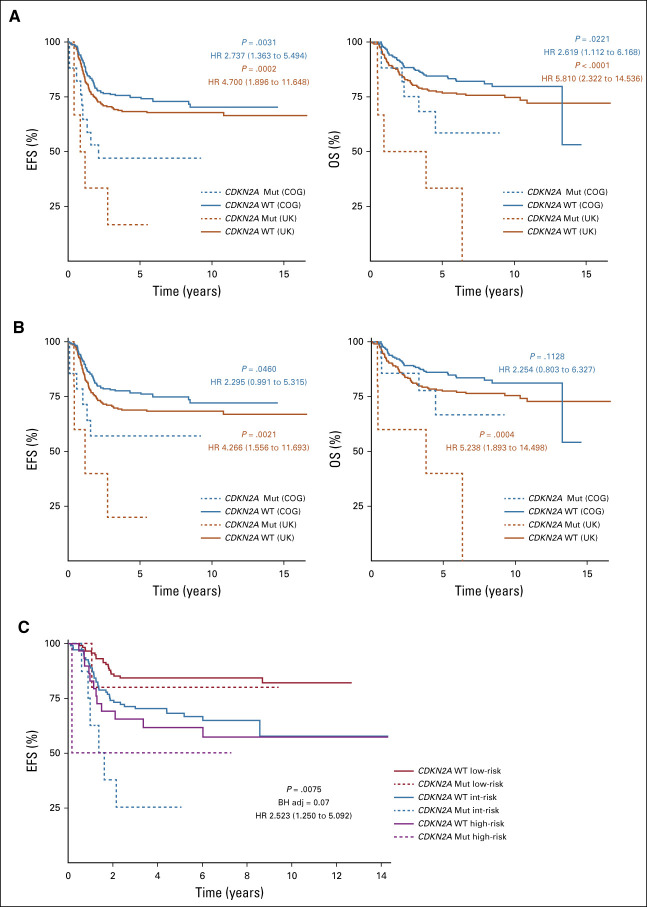
*CDKN2A* and association with survival. (A) EFS and OS by *CDKN2A* deletion in the COG cohort (blue) and the UK cohort (orange). (B) EFS and OS by *CDKN2A* status after removal of any cases with a co-occurring mutation of *MYOD1*: COG cohort (blue) and UK cohort (orange). (C) EFS within the COG cohort demonstrated that the observed effect of *CDKN2A* alteration was largely within the intermediate-risk group. BH adj, Benjamini-Hochberg–adjusted; COG, Children's Oncology Group; EFS, event-free survival; HR, hazard ratio; Mut, mutated; OS, overall survival; WT, wild type.

Mutations in the transcription factor *MYOD1* were found in 3% (n = 17 of 515) of all FN cases and no FP cases (Fig 4A). The observed mutations were confined to the previously reported hotspot codon change L122R.^25^ As noted, *MYOD1*-mutant tumors within this pediatric cohort occurred at an older mean age of 10.8 years (2.1-21.1 years) when compared with the rest of the cohort. Centrally reviewed pathology from COG and review of UK samples frequently noted spindle or sclerosing features of the tumor (Figs 4B1 and 4B2); however, interestingly, cases with densely packed cells that mimicked the dense pattern of embryonal rhabdomyosarcoma (ERMS) or RMS not otherwise specified were also found (Figs 4B3 and 4B4). EFS of those patients with *MYOD1* mutations within the COG cohort was dismal and associated with rapid progression in non–risk-stratified (EFS *P* < .0001; HR 6.839 [3.463-13.507]) and risk-stratified (EFS *P* < .0001; HR 5.579 [2.791-11.151]) analysis (Figs 4C and 4D). Parallel survival analysis of the UK cohort verified this observation in non–risk-stratified (EFS *P* = .0133; HR 3.320 [1.212-9.099]) and risk-stratified (EFS *P* = .0111; HR 3.455 [1.247-9.571]) analysis (Figs 4E and 4F). Importantly, within the *MYOD1*-mutant group, 23% of patients were identified before treatment as low-risk and 65% were identified as intermediate-risk. Consistent with previous reports,^26^ 53% of cases had a corresponding alteration of *PIK3CA* and *MYOD1* mutations were not found to be mutually exclusive with RAS mutations, with coexisting lesions seen in *NRAS* (n = 4), *HRAS* (n = 1), and *NF1* (n = 1). Interestingly, *MYOD1*-mutant tumors also frequently harbored deep deletions in *CDKN2A* (n = 4 of 17, 24%). Deep deletions or deleterious mutations of *CDKN2A* were present in 4% of all FN tumors and associated with a worse EFS (COG: *P* = .0031, HR 2.737 [1.363 to 5.494]; UK: *P* = .0031, HR 4.7 [1.896 to 11.648]) (Appendix Fig A7A, online only). This observation was independent of the co-occurrence of *MYOD1* mutations (Appendix Fig A7B), however was specific for cases within the intermediate-risk group (Appendix Fig A7C).

## DISCUSSION

Integration of molecular features into risk stratification and therapeutic decision making remains a major challenge to improving the care of any patient with a rare tumor. Decades of clinical trials led to the development of a complicated system for risk stratification of patients with RMS, on the basis of information from both the pretreatment tumor staging and surgical grouping.^4^ The imprecision of these assignments is known, which has important implications for how current therapy is delivered and how clinical trials incorporating novel agents are designed. Our international collaboration generated the largest cohort of clinically annotated and genomically characterized RMS tumors analyzed to date. The effort discovered critical genetic insights into the underpinnings of the disease and significant molecular markers that provide refinement to the current risk stratification of patients with RMS. On the basis of our results, we propose a new framework for the classification and treatment of RMS, using *TP53* and *MYOD1* mutations in addition to the *FOXO1* fusion status,^5-7^ which could be tested in prospective clinical trials (Appendix Table A4, online only).

The overall gene mutation frequency that was observed is consistent with previous sequencing studies^9,27^ with the notable exception of an increased frequency of tumor suppressor genes *TP53*, *NF1*, and *BCOR*. The observed increase in frequency of mutation of these genes likely results from improved depth of sequencing using a targeted assay approach. Of interest are the approximately 20% of FN tumors that have no driver mutation of a candidate gene. Beyond genome-wide aneuploidy and focal loss of heterozygosity of 11p15, the previous comprehensive analyses,^9,10,23,28^ and this focused analysis, have failed to discover recurrent genetic driver genes in this group of tumors. This suggests the need for continued comprehensive genomic and epigenetic evaluations that would allow identification of genes mutated at a low recurrence frequency, as well as alternative mechanisms of oncogenesis. The discovery that FN tumors are frequently driven by alteration of multiple coexisting mutations mirrors previous work that showed FN tumors are composed of multiple subclones that follow an evolutionary selection.^23^ This finding suggests that clonal evolution of these tumors may be significant, and elegant genomic work has highlighted how these processes might drive relapsed or refractory disease.^10^ Although the trend toward a worse outcome in patients with multiple mutations in the COG cohort is intriguing, this observation was not replicated in the UK cohort and may be confounded by the different therapeutic regimens that the patients received. Comprehensive, prospective assessment is required to address the validity of the survival correlations. In addition, studies designed to assay sequential tumor biopsies will be required to fully interrogate the mechanisms of metastasis and relapse in RMS.

Mutations in RAS isoforms have long been described as a driver of FN RMS.^29^ Our study clearly determines that the presence at diagnosis of a mutation in an RAS isoform or RAS pathway gene does not portend a poor prognosis, in contrast to previous smaller cohorts finding enrichment for RAS isoform mutations in high-risk cases.^10^ Although RAS was not found to be a prognostic predictor, we highlight an interesting observation that RAS isoform mutations appear to have some age-specific correlations, with *HRAS* occurring in the infants, *KRAS* occurring in the toddlers, and *NRAS* mutations with a peak in adolescence. Infants have previously been shown to have an inferior 5-year failure-free survival as compared with older patients (67% *v* 81%).^30^ This difference is attributed to the general reluctance to use more aggressive local control, including radiation, in these patients.^31^ Our results indicate that incorporation of targeted therapeutic agents such as tipifarnib (NCT04284774) or AMG510 (NCT03600883) may be particularly beneficial to this vulnerable and high-risk population. In addition, further mechanistic surveys of the developmental biology underlying these observations might have important implications for the generation of accurate preclinical models of RMS.

No molecular markers are currently used for risk stratification of FP RMS tumors. Amplification of the chromosomal regions 2p24 and 12q13-q15 and the implicated genes *MYCN* and *CDK4*, respectively, are the most recurrent lesions associated with a *FOXO1* fusion. Previous work dedicated to assigning the prognostic value of these lesions identified amplification of 12q13-q14, but not 2p24, as a marker of an aggressive subset of FP tumors.^32^ Other efforts discovered that in ARMS, overexpression or gain of genomic copies of *MYCN* was significantly associated with adverse outcome.^33^ The current study found inconsistent results for *MYCN* and *CDK4* amplification, with nonreproducible correlations noted between the two cohorts. There is evidence of a small subset of FP tumors that harbor a mutation of *TP53* at diagnosis and appear to be particularly aggressive. Ultimately, prospective consortium-level trials should include profiling of each of these genes to define their prognostic value and the biologic role they play in FP RMS.

From the seminal report of Li-Fraumeni syndrome,^34^ the role of *TP53* in ERMS oncogenesis has long been established; however, the association of *TP53* mutations with clinical outcome has previously been unknown. Given the lack of a corresponding germline sample, our study could not determine whether the discovered *TP53* mutation was germline or somatic. Despite this, we demonstrated that the presence of a *TP53* mutation was predictive of a worse outcome. This finding is consistent with reports from several cancer types that found mutation of *TP53* is associated with poor response and survival.^35^ This is also consistent with higher levels of TP53 protein in metastatic versus localized ERMS^36^ and also observations in zebra fish models of ERMS, where *tp53* mutations are linked to more aggressive and metastatic disease.^37^ Determination of *TP53* status in all cases of RMS therefore is critical, both for prognostic value and the implications that germline mutations have for genetic counseling.

*MYOD1* mutation of the L122R codon was reported by independent groups in 2014.^25,38^ *MYOD1*-mutant tumors make up only 3% of FN RMS, and this study highlights the importance of *MYOD1* mutations within the RMS population. These tumors have unique demographic, anatomic, and histologic characteristics, but none of these appear to definitively capture all *MYOD1*-mutant tumors. This suggests the need to incorporate sequencing of this gene into the diagnostic workup of FN RMS. Our observation that *MYOD1* mutations invariably co-occur with mutation in a second gene, most notably *PIK3CA* and *CDKN2A*, is consistent with a recent report.^26^ The co-occurrence with *CDKN2A* is of interest given that a recent large survey of soft tissue sarcomas of multiple histologies, including a small number of RMS tumors, implicated *CDKN2A* as a biomarker of poor prognosis.^39^ Although our study indicates that *CDKN2A* alterations may have prognostic value independent of *MYOD1*, this conclusion is based on low overall numbers. Therefore, we recommend that *CDKN2A* alterations be evaluated in prospective studies. Regardless, *MYOD1*-mutant tumors have an aggressive nature and have limited responses to current therapeutic regimens, which highlights the impetus to identify these cases and develop novel therapeutic trials for this rare subset of patients with RMS.

## Figures and Tables

**TABLE A1.**
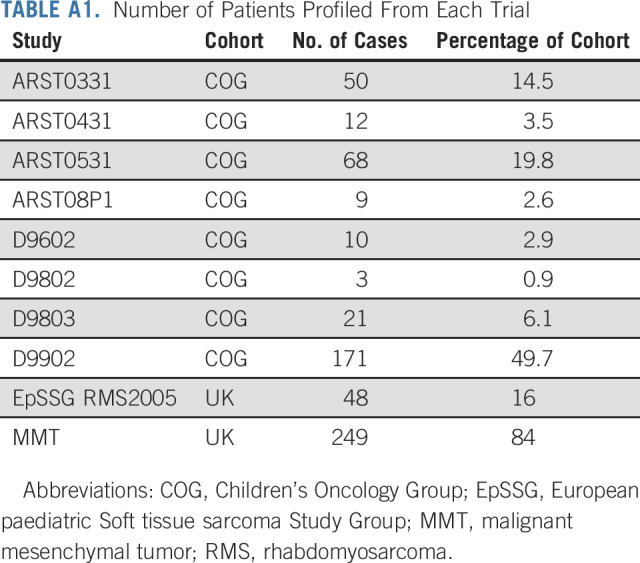
Number of Patients Profiled From Each Trial

**TABLE A2.**
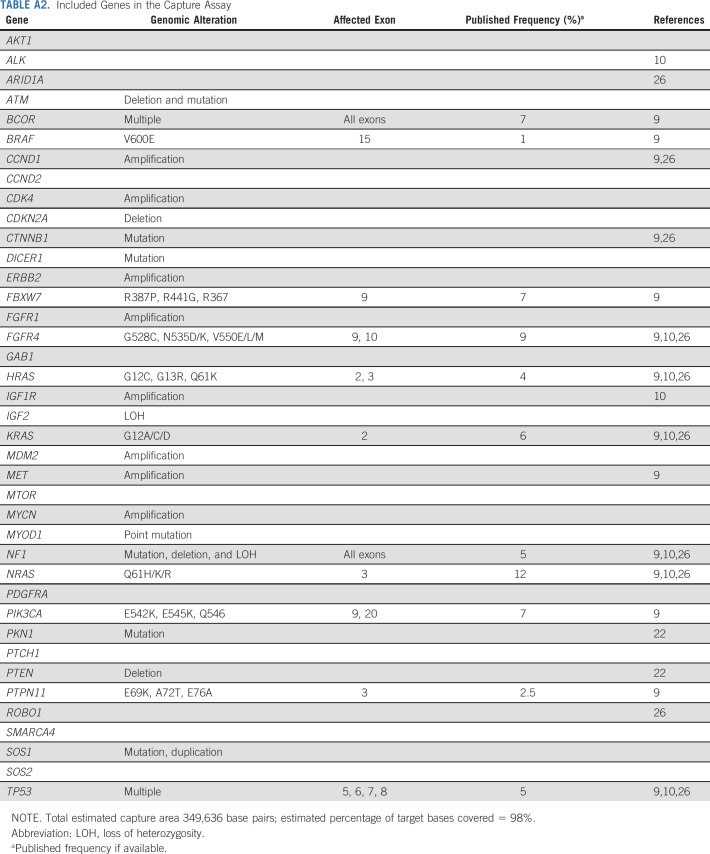
Included Genes in the Capture Assay

**TABLE A3.**
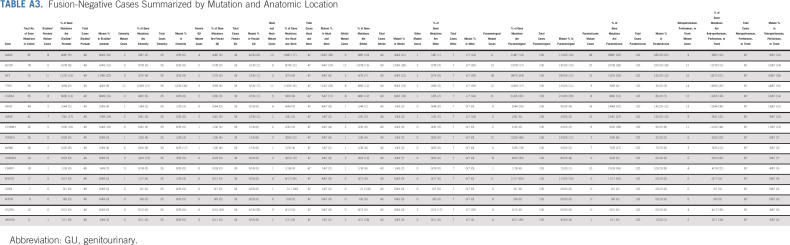
Fusion-Negative Cases Summarized by Mutation and Anatomic Location

**TABLE A4.**
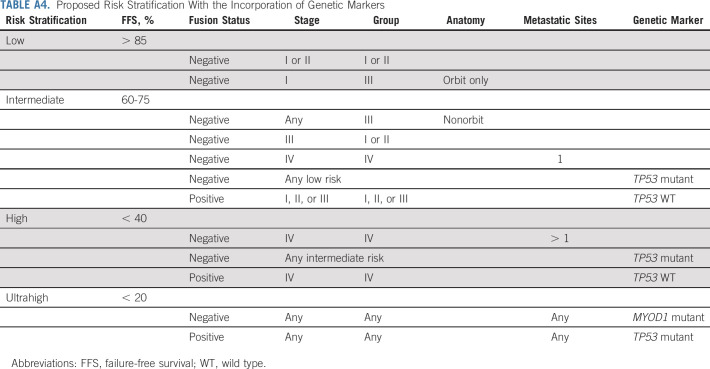
Proposed Risk Stratification With the Incorporation of Genetic Markers

## STUDY GROUPS

International Consortium: Children's Oncology Group; Children's Cancer and Leukaemia Group, Young Onset Sarcoma Subgroup; and the National Cancer Institute.
